# Non-linear phonon Peltier effect in dissipative quantum dot systems

**DOI:** 10.1038/s41598-018-23402-6

**Published:** 2018-03-26

**Authors:** Bitan De, Bhaskaran Muralidharan

**Affiliations:** Department of Electrical Engineering, Indian Institute of Technology Bombay, Powai, Mumbai, 400076 India

## Abstract

Solid state thermoelectric cooling is based on the electronic Peltier effect, which cools via an electronic heat current in the absence of an applied temperature gradient. In this work, we demonstrate that equivalently, a phonon Peltier effect may arise in the non-linear thermoelectric transport regime of a dissipative quantum dot thermoelectric setup described via *Anderson-Holstein* model. This effect leads to an electron induced phonon heat current in the absence of a thermal gradient. Utilizing the modification of quasi-equilibrium phonon distribution via charge induced phonon accumulation, we show that in a special case the polarity of the phonon heat current can be reversed so that setup can dump heat into the hotter reservoirs. In further exploring possibilities that can arise from this effect, we propose a novel charge-induced phonon switching mechanism that may be incited via electrostatic gating.

## Introduction

The study of zero-dimensional systems such as quantum dots is invaluable when it comes to fundamental aspects of nanoscale heat flow^1–4^. Significant interest has been triggered from the following motives: (a) Waste heat harvesting into electrical power^5–9^, (b) Solid state thermoelectric cooling^10,11^ and (c) Implementation of thermal energy in electronic logic design or *phonon computation*^12–16^. It is well known that electronic transport through such a quantum dot set up that is coupled to various phonon modes arising from the vibrational degrees of freedom gives rise to some unique features in charge transport^17–19^. A recent and notable work^20^ exploited the nanoscale interaction of charge and vibrational degrees of freedom via the use of electronic gating to selectively couple charge transport with phonon modes. This opens up new possibilities for optimizing thermoelectric performance^21^ as well as phonon switching linked to phonon computation.

Traditional solid-state thermoelectric cooling is based on the *electronic* Peltier effect that results from a change in the electronic energy current distribution accompanying electronic charge currents. In this work we show that phonon heat currents may as well be manipulated via electronic means in a dissipative quantum dot thermoelectric setup by what we call as the *phonon Peltier effect*. For this we utilize the interplay of charge and vibrational degrees of freedom in a quantum dot thermoelectric set up described via the *Anderson-Holstein* model.

**Figure 1:**
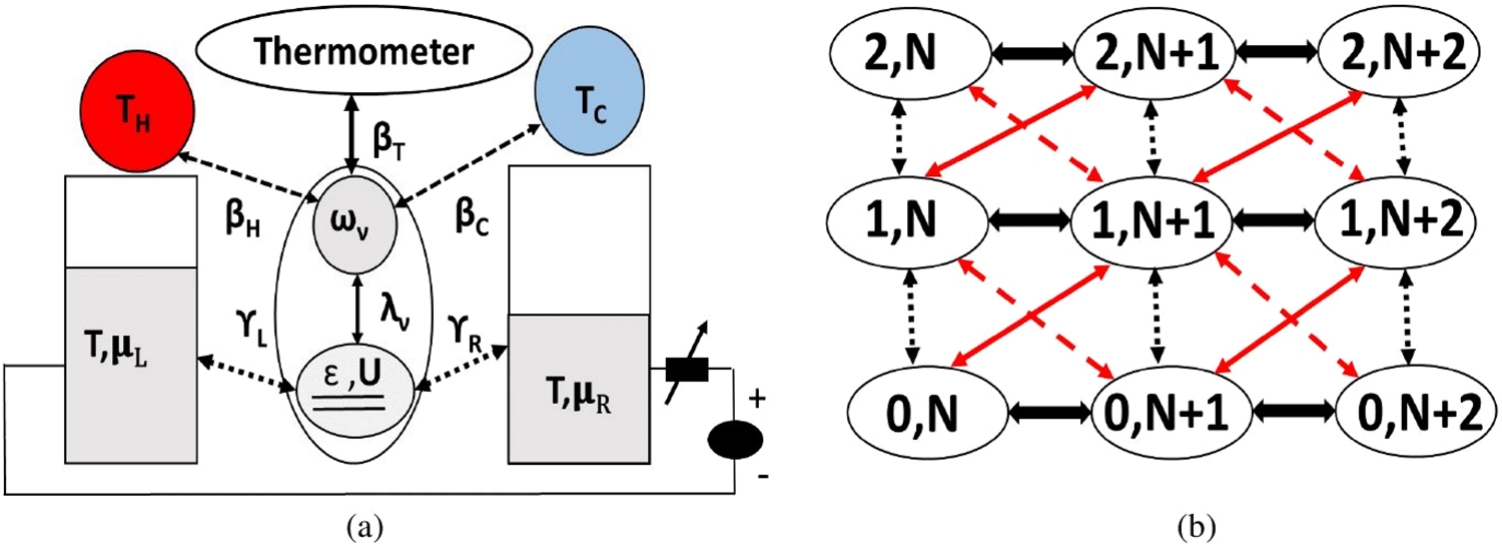
(**a**) Device Schematics: The quantum dot is weakly coupled to two electronic leads/contacts *L*, *R* described by their respective chemical potentials and phonon reservoirs *H*, *C* described by their respective temperatures. The corresponding electronic tunneling rate and phonon relaxation rate are ${\gamma }_{L(R)}$ and ${\beta }_{H(C)}$ respectively. Charge and phonon transport are coupled through electron-phonon interaction parameter *λ*, which drives the dot phonons out of equilibrium. The non-equilibrium phonon temperature is estimated using a thermometer bath weakly coupled to the dot. (**b**) The state transition diagram for electron-phonon many body Fock space. Each state is represented by $|n,q\rangle$, where *n* and *q* are the electron and phonon numbers of the state. The black dotted arrows represent direct electron tunneling processes, which do not change the dot phonon count. The red bold and dashed arrows represent phonon assisted tunneling processes which change the dot phonon count by +1 and −1 respectively. The black bold arrows represent reservoir assisted phonon transitions.

The most notable consequence of electron-phonon interaction is that the charge transport stimulated by an electrical voltage drives phonon away from equilibrium by modifying the phonon distribution^22–24^. The dynamics of such a charge induced phonon generation can be explored using the voltage controlled dissipative quantum dot set up described by *Anderson-Holestein* model^21,22,25–28^ considered in Fig. 1(a). A finite electron-phonon interaction modifies the phonon distribution and modulates the working temperature of the quantum dot, which can be monitored by a phonon thermometer weakly coupled to it^21,29^. In our earlier work^21^, we explored how charge-phonon interplay can influence the power-efficiency trade-off thereby influencing the thermoelectric operation. In this paper, we theoretically demonstrate how a voltage bias rather than a temperature gradient excites a phonon heat current when the same charge-phonon interplay modulates the quasi-equilibrium phonon distribution. In the present paper, we explain the relevant physics that describes the nature of phonon heat flow at different operating points and investigate the role of charge-phonon interplay in this context. We justify the mechanism of *phonon Peltier* effect by establishing an in-built phonon thermal gradient between the dot and the phonon reservoirs by coupling it to a phonon thermometer^21^. We then investigate the role of the temperature of the macroscopic phonon reservoirs in controlling the non-equilibrium phonon distribution, and hence the polarity of phonon flow. Based on this we address possible areas of practical interest that can arise out of this effect to propose a charge induced phonon switch.

## Theoretical Formulation

### Model Hamiltonian

The set up depicted in Fig. 1(a) comprises a quantum dot described by the dissipative *Anderson-Holestein* model. Charge and phonon currents are set up by applying a voltage and thermal bias across the metallic electrodes ${\alpha }_{1}$ (${\alpha }_{1}\in L,R$) and the phonon reservoirs ${\alpha }_{2}$ (${\alpha }_{2}\in H,C$) respectively. The composite system Hamiltonian, $\hat{H}$, is the sum of the Hamiltonians of the dot (${\hat{H}}_{D}$), the electrodes (${\hat{H}}_{{\alpha }_{1}}$), the reservoirs (${\hat{H}}_{{\alpha }_{2}}$) and the tunneling part (${\hat{H}}_{T}$). The dot Hamiltonian is described as

1 $$\begin{array}{c}{\hat{H}}_{D}=(\sum _{{\rm{\sigma }}}\varepsilon {d}_{{\rm{\sigma }}}^{\dagger }{d}_{{\rm{\sigma }}}+U{d}_{\uparrow }^{\dagger }{d}_{\uparrow }{d}_{\downarrow }^{\dagger }{d}_{\downarrow })+\hslash {\omega }_{{\rm{\nu }}}{\hat{b}}_{{\rm{\nu }}}^{\dagger }{\hat{b}}_{{\rm{\nu }}}+\sum _{{\rm{\sigma }}}{\lambda }_{{\rm{\nu }}}\hslash {\omega }_{{\rm{\nu }}}{d}_{{\rm{\sigma }}}^{\dagger }{d}_{{\rm{\sigma }}}({\hat{b}}_{{\rm{\nu }}}^{\dagger }+{\hat{b}}_{{\rm{\nu }}}),\end{array}$$

where the electronic part of the dot consists of a single spin degenerate energy level with energy *ε* and a Coulomb interaction energy *U*. The phonon part represents a single phonon mode with frequency ${\omega }_{\nu }$. Inside the dot, the electrons and phonons interact through a dimensionless coupling parameter, ${\lambda }_{\nu }$. Here, ${d}_{\sigma }^{\dagger }({d}_{\sigma })$($\sigma \in \uparrow ,\downarrow$) and ${b}_{\nu }^{\dagger }({b}_{\nu })$ denote the creation (annihilation) operator of the dot electrons and phonons respectively.

The contact Hamiltonian ${H}_{{\alpha }_{1}}$ represents a reservoir of non-interacting and spin degenerate electrons in the momentum eigenstate representation $k{\rm{\sigma }}^{\prime}$ with eigen energies ${\varepsilon }_{k{\rm{\sigma }}^{\prime} }$. On the other hand, the reservoir Hamiltonian ${H}_{{\alpha }_{2}}$ characterizes a bath of independent phonon modes *r* with frequencies ${\omega }_{{\alpha }_{2}r}$. They are given by

2 $${\hat{H}}_{\alpha 1}=\sum _{{\alpha }_{1}\in L,R}\sum _{k\sigma ^{\prime} }{\varepsilon }_{{\alpha }_{1}k{\rm{\sigma }}^{\prime} }{\hat{c}}_{{\alpha }_{1}k{\rm{\sigma }}^{\prime} }^{\dagger }{\hat{c}}_{{\alpha }_{1}k{\rm{\sigma }}^{\prime} },$$

3 $${\hat{H}}_{{\alpha }_{2}}=\sum _{{\alpha }_{2}\in H,C}\sum _{r}\hslash {\omega }_{{\alpha }_{2}r}{\hat{B}}_{{\alpha }_{2}r}^{\dagger }{\hat{B}}_{{\alpha }_{2}r}$$

Here, ${\hat{c}}_{{\alpha }_{1}k{\rm{\sigma }}^{\prime} }^{\dagger }({\hat{c}}_{{\alpha }_{1}k{\rm{\sigma }}^{\prime} })$ creates (annihilates) an electron with momentum *k* and spin ${\rm{\sigma }}^{\prime} \in \uparrow ,\downarrow$ in the contacts ${\alpha }_{1}\in L,R$. Similarly ${\hat{B}}_{{\alpha }_{2}r}^{\dagger }({\hat{B}}_{{\alpha }_{2}r})$ creates(annihilates) a phonon mode *r* in the reservoirs ${\alpha }_{2}\in H,C$ of energies $\hslash {\omega }_{{\alpha }_{2}r}$. Electronic tunneling processes between the dot and the contacts and the phonon relaxation processes from the dot to the reservoirs are described by the Hamiltonian ${\hat{H}}_{T}$, which is defined as

4 $${\hat{H}}_{T}=\sum _{k{\rm{\sigma }}^{\prime} ,{\rm{\sigma }}}[{\tau }_{{\alpha }_{1}}^{el}{\hat{c}}_{{\alpha }_{1}k{\rm{\sigma }}^{\prime} }^{\dagger }{\hat{d}}_{{\rm{\sigma }}}+H\mathrm{.}c\mathrm{.]}+\sum _{\nu ,{\alpha }_{2}r}{\tau }_{{\alpha }_{2}}^{ph}({\hat{B}}_{{\alpha }_{2}r}^{\dagger }+{\hat{B}}_{{\alpha }_{2}r})({\hat{b}}_{{\rm{\nu }}}^{\dagger }+{\hat{b}}_{{\rm{\nu }}}),$$

where the spin independent coupling energy between the dot and the contact electrons is ${\tau }_{{\alpha }_{1}}^{el}$ and the mode independent coupling energy between the dot and the reservoir phonons is ${\tau }_{{\alpha }_{2}}^{ph}$.

The diagonalization of the dot Hamiltonian is carried out through the standard polaron transformation which leads to the renormalization of the on-site energy ($\tilde{\varepsilon }=\varepsilon -{\lambda }_{{\rm{\nu }}}^{2}\hslash {\omega }_{{\rm{\nu }}}$), and the Coulomb interaction term ($\tilde{U}=U-2{\lambda }_{{\rm{\nu }}}^{2}\hslash {\omega }_{{\rm{\nu }}}$). The eigenstates of the renormalized dot Hamiltonian are denoted as $|n,q\rangle$, where *n* and *q* are the electron and phonon numbers of the states. The energy eigen values of those states become ${E}_{(n,q)}={\tilde{E}}_{n}+q\hslash {\omega }_{{\rm{\nu }}}$, where, ${\tilde{E}}_{0}\mathrm{=0,}\,{\tilde{E}}_{1}=\tilde{\varepsilon },{\tilde{E}}_{2}=2\tilde{\varepsilon }+\tilde{U}$, corresponding to the *n* = 0, 1 and 2 electron number spaces respectively. Polaron transformation also leads to the renormalization of electron coupling energies ${\tilde{\tau }}_{{\alpha }_{1}}^{el}={\tau }_{{\alpha }_{1}}^{el}exp[-{\lambda }_{{\rm{\nu }}}({\hat{b}}_{{\rm{\nu }}}-{\hat{b}}_{{\rm{\nu }}}^{\dagger })]$. The modification of phonon coupling energy ${\tau }_{{\alpha }_{2}}^{ph}$ is neglected considering the weak coupling between the dot and the reservoir phonons. Using these, the rate of electron tunneling ${\gamma }_{{\alpha }_{1}}$ and phonon relaxation ${\beta }_{{\alpha }_{2}}$ are evaluated using the Fermi’s golden rule: ${\gamma }_{{\alpha }_{1}}=\frac{2\pi }{\hslash }{\sum }_{{\alpha }_{1}}|{\tilde{\tau }}_{{\alpha }_{1}}^{el}{|}^{2}{\rho }_{{\alpha }_{1}{\rm{\sigma }}^{\prime} }$ and ${\beta }_{{\alpha }_{2}}=\frac{2\pi }{\hslash }|{\tau }_{{\alpha }_{2}}^{ph}{|}^{2}{D}_{{\alpha }_{2}}$. Here ${\rho }_{{\alpha }_{1}{\rm{\sigma }}^{\prime} }$ and ${D}_{{\alpha }_{2}}$ are the constant electron and phonon density of states associated with electrodes ${\alpha }_{1}$ and reservoirs *α*_2_.

### Transport equations

We first describe physical implications imposed by our setup which leads to the current formulation of the transport equations. First, we use the approximation $\hslash {\gamma }_{{\alpha }_{1}}\gg \hslash {\beta }_{{\alpha }_{2}}$, so that we can ignore the system damping^30^ and assume that each reservoir generates phonon currents independent of other reservoirs^31–33^. Second, we also set $\hslash {\omega }_{{\rm{\nu }}}\gg \hslash {\gamma }_{{\alpha }_{1}}$, so that any overlap between two adjacent phonon sidebands is excluded and subsequently, the electron tunneling events are completely uncorrelated^21,34,35^. This assumption gives us enough scope to ignore phonon reservoir memory. Lastly, the sequential tunneling limit is assumed such that ${k}_{B}T\gg \hslash {\gamma }_{{\alpha }_{1}},\hslash {\beta }_{{\alpha }_{2}}$, where, charge and heat transport are described via the master equation framework by evaluating the steady state probability ${P}_{(n,q)}$ of an electron-phonon many-body state $|n,q\rangle$^36–39^. The electronic tunneling between the dot and the electrodes (${\alpha }_{1}\in L,R$) causes transitions between two electron-phonon states,$|n,\,q\rangle$ and $|n\pm \mathrm{1,}\,q^{\prime} \rangle$, with the following rates:

5 $$\begin{array}{c}{R}_{(n,q)\to (n+\mathrm{1,}q^{\prime} )}=\sum _{{\alpha }_{1}\in L,R}{\gamma }_{{\alpha }_{1}}|\langle n,q|{\hat{\tilde{d}}}_{{\rm{\sigma }}}|n+\mathrm{1,}\,q^{\prime} \rangle {|}^{2}\times {f}_{{\alpha }_{1}}(\frac{{E}_{n+\mathrm{1,}q^{\prime} }-{E}_{n,q}-{\mu }_{{\alpha }_{1}}}{{k}_{B}{T}_{{\alpha }_{1}}}),\end{array}$$

6 $$\begin{array}{c}{R}_{(n,q)\to (n-\mathrm{1,}q^{\prime} )}=\sum _{{\alpha }_{1}\in L,R}{\gamma }_{{\alpha }_{1}}|\langle n,q|{\hat{\tilde{d}}}_{{\rm{\sigma }}}^{\dagger }|n-\mathrm{1,}\,q^{\prime} \rangle {|}^{2}\times [1-{f}_{{\alpha }_{1}}(\frac{{E}_{n+\mathrm{1,}q^{\prime} }-{E}_{n,q}-{\mu }_{{\alpha }_{1}}}{{k}_{B}{T}_{{\alpha }_{1}}})]\end{array}$$

where ${\tilde{d}}_{{\rm{\sigma }}}^{\dagger }$(${\tilde{d}}_{{\rm{\sigma }}}$) is the renormalized creation(annihilation) operator for dot electrons following the polaron transformation. Similarly, the rate of phonon transition between the dot and reservoirs (${\alpha }_{2}\in H,C$) causes a transition between the states $|n,q\rangle$ and $|n,q\pm 1\rangle$, with the following rates that abide by the Boltzman ratio:

7 $$\begin{array}{c}{R}_{(n,q)\to (n,q+\mathrm{1)}}=\sum _{{\alpha }_{2}\in H,C}{\beta }_{{\alpha }_{2}}(q+\mathrm{1)}exp(-\frac{\hslash {\omega }_{{\rm{\nu }}}}{{k}_{B}{T}_{{\alpha }_{2}}}),\end{array}$$

8 $$\begin{array}{c}{R}_{(n,q)\to (n,q-\mathrm{1)}}=\sum _{{\alpha }_{2}\in H,C}{\beta }_{{\alpha }_{2}}(q+\mathrm{1)}\end{array}$$

Then the master equation for the probabilities ${P}_{n,q}$ of an electron-phonon state $|n,q\rangle$ can be written as follows:

9 $$\begin{array}{c}\frac{d{P}_{(n,q)}}{dt}=\sum _{q^{\prime} }[{R}_{(n\pm \mathrm{1,}q^{\prime} )\to (n,q)}^{el}{P}_{(n\pm \mathrm{1,}q^{\prime} )}-{R}_{(n,q)\to (n\pm \mathrm{1,}q^{\prime} )}^{el}{P}_{(n,q)}]+[{R}_{(n,q\pm \mathrm{1)}\to (n,q)}^{ph}{P}_{(n,q\pm \mathrm{1)}}-{R}_{(n,q)\to (n,q\pm \mathrm{1)}}^{ph}{P}_{(n,q)}\mathrm{].}\end{array}$$

In the steady state $\frac{d{P}_{(n,q)}}{dt}$ becomes $0$ and we can evaluate the steady state probabilities ${P}_{n,q}$, using which we can calculate the charge and heat currents associated with the electrodes ${\alpha }_{1}\in L,R$ and the reservoirs ${\alpha }_{2}\in H,C$ as

10 $$\begin{array}{c}{I}_{{\alpha }_{1}}=\sum _{n,q}\sum _{n,q^{\prime} }-q[{R}_{(n^{\prime} ,q^{\prime} )\to (n,q)}^{e{l}_{{\alpha }_{1}}}{P}_{(n^{\prime} ,q^{\prime} )}-{R}_{(n,q)\to (n^{\prime} ,q^{\prime} )}^{e{l}_{{\alpha }_{1}}}{P}_{(n^{\prime} ,q^{\prime} )}]\delta (n\pm \mathrm{1,}\,n^{\prime} ),\end{array}$$

11 $$\begin{array}{c}{I}_{p{h}_{{\alpha }_{2}}}^{Q}=\sum _{n,q}\sum _{n,q^{\prime} }\hslash {\omega }_{{\rm{\nu }}}[{R}_{(n,q)\to (n^{\prime} ,q^{\prime} )}^{p{h}_{{\alpha }_{2}}}{P}_{(n,q)}-{R}_{(n^{\prime} ,q^{\prime} )\to (n,q)}^{p{h}_{{\alpha }_{2}}}{P}_{(n^{\prime} ,q^{\prime} )}]\delta (n,n^{\prime} )\delta (q\pm \mathrm{1,}\,q^{\prime} \mathrm{).}\end{array}$$

Since the electron tunneling rate ${\gamma }_{{\alpha }_{1}}$ is greater than the phonon relaxation rate ${\beta }_{{\alpha }_{2}}$, the steady state probabilities ${P}_{n,q}$ and hence the phonon heat current ${I}_{p{h}_{{\alpha }_{2}}}^{Q}$ are primarily determined by the charge transport. However the electron-phonon interaction parameter ${\lambda }_{{\rm{\nu }}}$ plays an important role to modify the electronic tunneling rate ${\gamma }_{{\alpha }_{1}}$ to be discussed in detail in the following section.

### Inter-coupled electron and phonon transport

We note that in the presence of electron-phonon interaction, the effective electron tunneling rate ${\gamma }_{{\alpha }_{1}}^{eff}$ between the two states $(n,q)$ and $(n\pm \mathrm{1,}\,q^{\prime} )$ is modified by the ${\lambda }_{{\rm{\nu }}}$ dependent *Frank-Condon*^40–44^ overlapping factor($F{C}_{q,q^{\prime} }$) between them, and it is denoted as

12 $$\begin{array}{c}{\gamma }_{{\alpha }_{1}}^{eff}={\gamma }_{{\alpha }_{1}}[|\langle n,q|{\hat{\tilde{d}}}_{{\rm{\sigma }}}^{\dagger }|n^{\prime} ,q^{\prime} \rangle {|}^{2}\delta (n^{\prime} ,n-\mathrm{1)}+|\langle n,q|{\hat{\tilde{d}}}_{{\rm{\sigma }}}|n+1,q^{\prime} \rangle {|}^{2}\delta (n^{\prime} ,n+\mathrm{1)]}\\ {\gamma }_{{\alpha }_{1}}^{eff}={\gamma }_{{\alpha }_{1}}{|F{C}_{q,q^{\prime} }|}^{2}\\ {|F{C}_{q,q^{\prime} }|}^{2}=exp(-{\lambda }_{{\rm{\nu }}}^{2})\frac{k!}{K!}{\lambda }^{\mathrm{2(}K-k)}{[{L}_{k}^{K-k}({\lambda }_{{\rm{\nu }}}^{2})]}^{2},\end{array}$$

where ${L}_{k}^{K-k}$ is the associated Laguerre polynomial with $k=min(q,q^{\prime} )$ and $K=max(q,q^{\prime} )$. When $q\ne q^{\prime}$ the charge transport leads to phonon generation (or absorption) in the dot with a rate $G{E}_{ph}^{{\alpha }_{1}}={\sum }_{n,q}{\sum }_{n\pm \mathrm{1,}q^{\prime} }(q^{\prime} -q){P}_{(n,q)}{R}_{(n,q)\to (n\pm \mathrm{1,}p^{\prime} )}^{{\alpha }_{1}}$. One should note from (12), that when ${\lambda }_{{\rm{\nu }}}=0$, ${\gamma }_{{\alpha }_{1}}^{eff}$ becomes zero unless $q=q^{\prime}$. It implies that there is no charge assisted phonon generation in the absence of electron-phonon interaction. The generated phonons are further removed by the reservoirs with a rate of $R{E}_{ph}^{{\alpha }_{2}}$ given by^23^:

13 $$\begin{array}{c}R{E}_{ph}^{{\alpha }_{2}}={\beta }_{{\alpha }_{2}}\frac{\langle {N}_{ph}\rangle -\langle {N}_{ph}^{eq}\rangle }{1+\langle {N}_{ph}^{eq}\rangle }\mathrm{.}\end{array}$$

In the above equation, $\langle {N}_{ph}^{eq}\rangle =[exp(\frac{\hslash \omega }{{k}_{B}{T}_{{\alpha }_{2}}})-{\mathrm{1]}}^{-1}$ is the average equilibrium phonon number of the reservoirs. The average dot phonon occupation is defined as $\langle {N}_{ph}\rangle ={\sum }_{n,q}q{P}_{(n,q)}$. When the system is in equilibrium (${\lambda }_{{\rm{\nu }}}=0$), $\langle {N}_{ph}\rangle$ equals to $\langle {N}_{ph}^{eq}\rangle$ due to no charge assisted phonon generation and $R{E}_{ph}^{{\alpha }_{2}}$ nullifies and we get zero ${I}_{p{h}_{{\alpha }_{2}}}^{Q}$. In this case, the phonon distributions obey the following Boltzman’s ratio^45^:

14 $$\begin{array}{c}\frac{{P}_{(n,q+\mathrm{1)}}}{{P}_{(n,q)}}=exp[-(\frac{\hslash {\omega }_{\nu }}{{k}_{B}{T}_{{\alpha }_{2}}})]\end{array}$$

On the other way, in the non-equilibrium condition (${\lambda }_{{\rm{\nu }}}\ne 0$), $G{E}_{ph}^{{\alpha }_{1}}$ becomes finite. If $G{E}_{ph}^{{\alpha }_{1}}$ exceeds $R{E}_{ph}^{{\alpha }_{2}}$, phonons accumulate in the dot and the dot temperature deviates from the reservoir temperature. The expression for the dot temperature ${T}_{M}$, can be approximated from the Boltzmann’s ratio with a quasi-equilibrium assumption as^46^:

15 $${T}_{M}=\frac{\hslash {\omega }_{{\rm{\nu }}}}{{k}_{B}}{[ln(\frac{{P}_{n,q}}{{P}_{n,q+1}})]}^{-1}$$

The charge transport assisted phonon transport gives rise to some interesting outcomes which we are going to elaborate now. From now on, we will denote ${\lambda }_{{\rm{\nu }}}$, ${I}_{{\alpha }_{1}}$, ${I}_{p{h}_{{\alpha }_{2}}}^{Q}$, $G{E}_{ph}^{{\alpha }_{1}}$, $R{E}_{ph}^{{\alpha }_{2}}$ as $\lambda$, $I$, ${I}_{Q}^{ph}$, ${G}_{ph}$, ${R}_{ph}$, respectively, to maintain notational simplicity. Throughout the work, we will maintain the fact that no thermal gradient is applied across the electrodes (i.e ${T}_{L}={T}_{R}=T$) as well as across the reservoirs (i.e ${T}_{H}={T}_{C}={T}_{B}$). We also assume a symmetric electronic couping between the dot and the electrodes (${\gamma }_{L}={\gamma }_{R}=\gamma$) and a symmetric phonon coupling between the dot and reservoirs (${\beta }_{H}={\beta }_{C}=\beta$).

## Results

### Voltage stimulated phonon heat current

**Figure 2:**
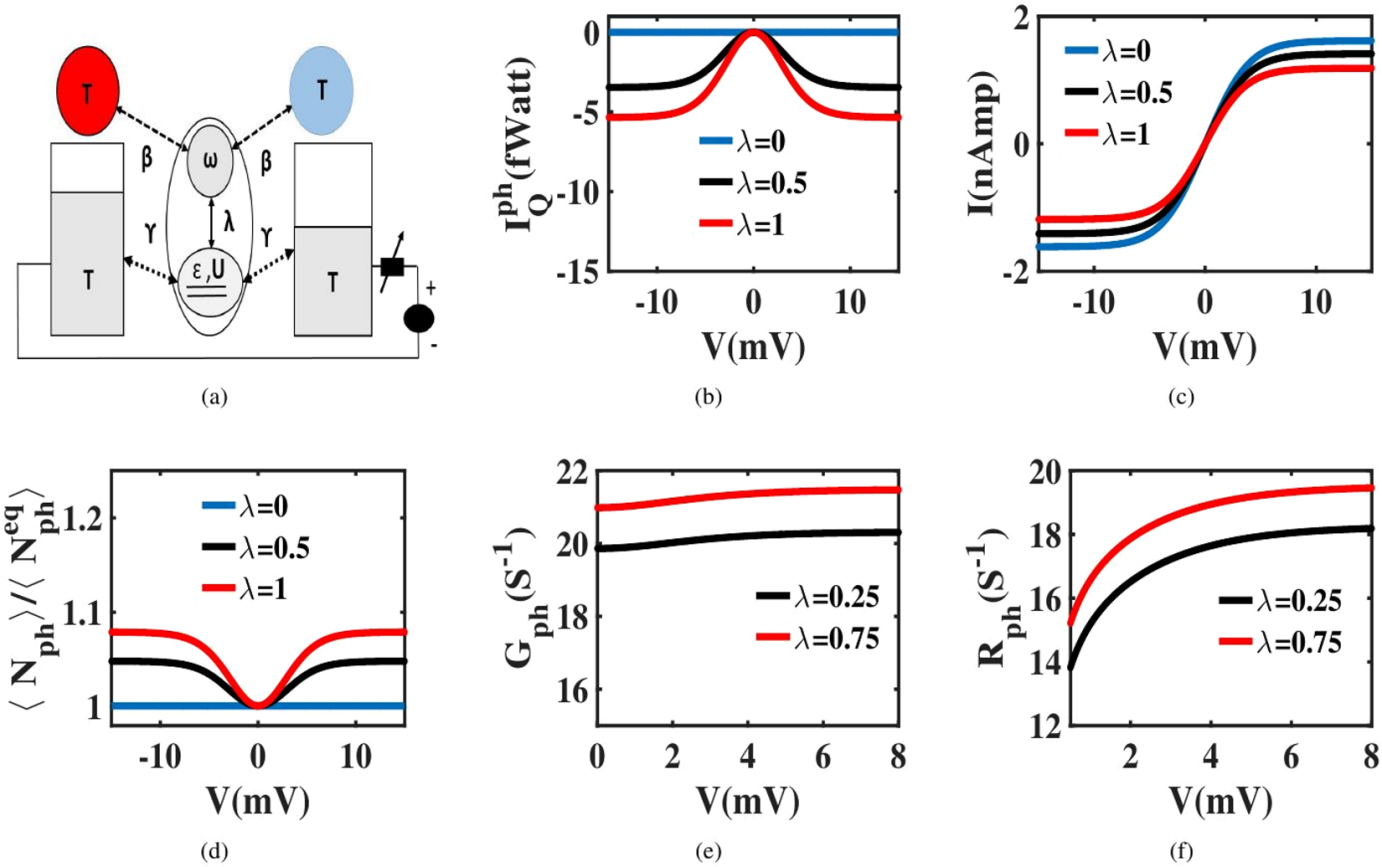
Voltage induced phonon transport. (**a**) Modified device schematic: Electrodes and reservoirs are maintained at same temperature (i.e ${T}_{L}={T}_{R}={T}_{H}={T}_{C}=T$). The dot is symmetrically electronic coupled to the electrodes and symmetrically phonon coupled to the reservoirs (i.e ${\gamma }_{L}={\gamma }_{R}=\gamma$ and ${\beta }_{H}={\beta }_{C}=\beta$). (**b**) Variation of ${I}_{Q}^{ph}$ with voltage for different $\lambda$ keeping ${\rm{\Delta }}{T}_{ph}=0$. ${I}_{Q}^{ph}$ increases with voltage and ultimately saturates at high voltage along with the charge current. (**c**) The voltage dependence of charge current. (**d**) Deviation of $\langle {N}_{ph}\rangle$ from $\langle {N}_{ph}^{eq}\rangle$ as voltage increases for non-zero $\lambda$. This gives rise to the voltage dependence of ${I}_{Q}^{ph}$. (**e**) The variation of ${G}_{ph}$(in log-scale) with voltage for different values of $\lambda$. (**f**) The variation of ${R}_{ph}$(in log-scale) with voltage for different values of $\lambda$. Since ${R}_{ph}$ is always lesser as compared to ${G}_{ph}$, $\langle {N}_{ph}\rangle$ dominates over $\langle {N}_{ph}^{eq}\rangle$.

We start by investigating the voltage response of ${I}_{Q}^{ph}$ considering that the electrodes and reservoirs are at same temperature (${T}_{B}=T$) as shown in Fig. 2(a). Figure 2(b), notes that in equilibrium condition (${\lambda }_{{\rm{\nu }}}=0$), the phonon heat current ${I}_{Q}^{ph}$ vanishes for all voltages. It is consistent with the fact that in the absence of electron-phonon interaction, charge assisted phonon generation freezes and hence, the dot phonons remain in equilibrium with the reservoir phonons. This leads to the nullification of ${R}_{ph}$ as well as ${I}_{Q}^{ph}$. The scenario changes when we turn on, a finite electron-phonon interaction ($\lambda \ne 0$), where a non-trivial voltage dependence of ${I}_{Q}^{ph}$ results, as seen in Fig. 2(b). In this case, ${I}_{Q}^{ph}$ first shoots up and then levels off as voltage increases. When voltage becomes large, higher order phonon assisted tunneling events are suppressed due to limited *Frank-condon* overlap^47^. This leads to the saturation of both $I$ and ${G}_{ph}$. Consequently, ${I}_{Q}^{ph}$ also levels off at large voltage.

As explained earlier, a finite *λ* facilitates phonon generation and the average dot phonon distribution $\langle {N}_{ph}\rangle$ deviates from ${N}_{ph}^{eq}$ as shown in Fig. 2(d). Hence, as dictated by (13), the phonon removal rate changes with voltage ultimately leading to the voltage dependence of ${I}_{Q}^{ph}$. Throughout this work we maintain ${\gamma }_{L(R)} > {\beta }_{H(C)}$, resulting in *G*_*ph*_ exceeding *R*_*ph*_ as noticed in Fig. 2(e) and (f). It leads to a phonon accumulation inside the dot with $\langle {N}_{ph}\rangle$ always exceeding $\langle {N}_{ph}^{eq}\rangle$. As a result, ${I}_{Q}^{ph}$ remains negative, i.e., ${I}_{Q}^{ph}$ flows away from the dot toward the reservoirs. From this discussion it can be concluded that the voltage dependence of ${I}_{Q}^{ph}$ primarily depends on $\langle {N}_{ph}^{eq}\rangle$, which is a function of $\lambda$ and voltage.

An important point to be noted is that the variation of ${I}_{Q}^{ph}$ takes place only in the non-linear voltage range, i.e., when $q{V}_{app}\gg {k}_{B}T$^48^. In the linear response regime, phonon current abides with the *Onsager’s reciprocity*^49,50^. The voltage dependence of ${I}_{Q}^{ph}$ immediately hints at a physically new effect which can be thought of as the *phonon Peltier effect* rather than the conventional *electronic Peltier effect*.

### Phonon Peltier effect

**Figure 3:**
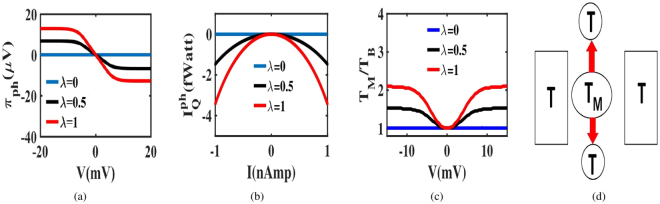
Non-linear phonon peltier effect. (**a**) Plots on the variation of ${\pi }_{ph}$ with voltage for different *λ*. (**b**) The variation of ${I}_{Q}^{ph}$ with the charge current *I*. The Peltier coefficient, ${\pi }_{ph}$ is extracted from the gradient of this plot.(**c**) The deviation of dot temperature *T*_*M*_ from reservoir temperature *T*_*B*_ (here ${T}_{B}=T$) with voltage for different values of *λ*. Temperature gradient between the dot and the reservoirs is confirmed when *λ* becomes non-zero thus validating the phonon Peltier effect. (**d**) A schematic of phonon heat flow as a consequence of the Peltier effect.

The phonon Peltier coefficient is calculated as ${\pi }_{ph}={I}_{Q}^{ph}/I$, provided there is no temperature gradient (i.e., ${T}_{H}={T}_{C}$). Here we maintain the analogy with electronic Peltier coefficient ${\pi }_{el}={I}_{Q}^{el}/I$. The variation of ${\pi }_{ph}$ is plotted as a function of voltage in Fig. 3(a). In our simulations, we plot ${\pi }_{ph}=d{I}_{Q}^{ph}/dI$ to avoid the discontinuity at the short-circuit (${V}_{SC}=0$) point and ${\pi }_{ph}$ is extracted from the gradient of the ${I}_{Q}^{ph}-I$ plot shown in the inset of Fig. 3(a). We immediately note that ${\pi }_{ph}$ vanishes when there is no *λ*. When *λ* becomes finite, ${\pi }_{ph}$ increases almost linearly within a small voltage range and levels off at large voltages along with the charge current *I*. Typically, the Peltier effect causes a temperature gradient when subject to an electrical excitation. In this case, we note a temperature difference is created between the dot and the reservoir phonons, which we estimate via the dot temperature *T*_*M*_ using a thermometer probe^21,51^. Figure 3(b) shows a clear deviation of *T*_*M*_ from the reservoir temperature *T*_*B*_. Since ${\gamma }_{{\alpha }_{1}} > {\beta }_{{\alpha }_{2}}$, phonons accumulate in the dot and hence *T*_*M*_ is always greater than *T*_*B*_. Consequently, the phonon current will flow away from the dot as shown in the inset of Fig. 3(b) and signifies Peltier effect.

### Charge controlled phonon distribution

**Figure 4:**
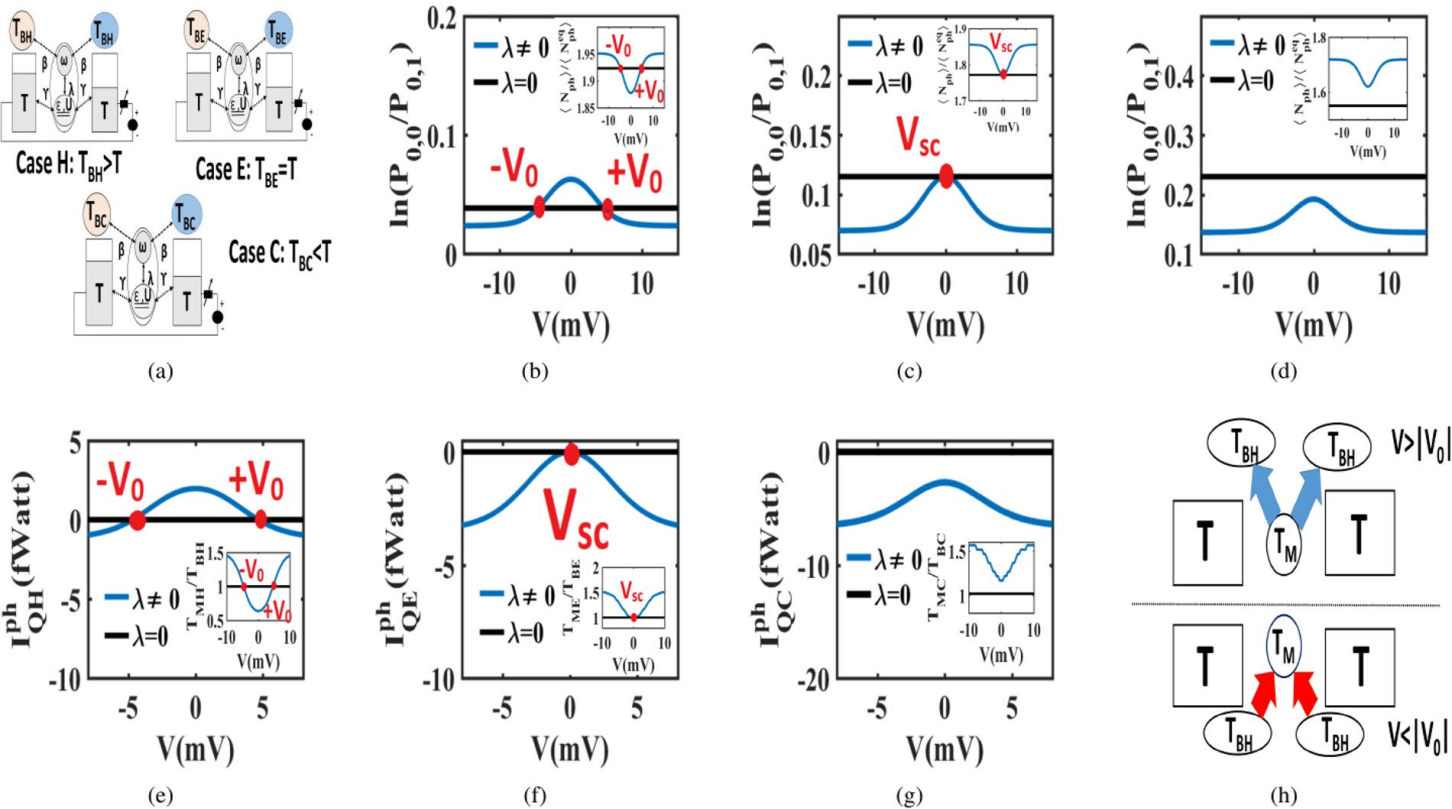
Manipulation of phonon equilibrium distribution by electron flow. (**a**) We consider three cases: (i) **Case H**: Reservoir temperature ${T}_{B}={T}_{BH}$ greater than the electrode temperature *T*, (ii) **Case E**: Reservoir temperature *T*_*B*_ = *T*_*BE*_ equal to the electrode temperature *T* and (iii) **Case C**: Reservoir temperature ${T}_{B}={T}_{BC}$ less than the electrode temperature *T*. (**b**,**c** and **d**) show the voltage dependence of the phonon probability distribution under non-equilibrium ($\lambda \ne 0$) and equilibrium ($\lambda =0$) conditions for Cases H, E, and C respectively. Insets present the respective deviations of $\langle {N}_{ph}\rangle$ from $\langle {N}_{ph}^{eq}\rangle$. (**e**,**f** and **g**) depict the voltage dependence of ${I}_{Q}^{ph}$ under non-equilibrium ($\lambda \ne 0$) and equilibrium ($\lambda =0$) conditions for Cases H, E, C respectively. Each profile of ${I}_{Q}^{ph}$ is consistent with the temperature gradient between the dot and reservoir as shown in the insets of (**e**,**f** and **g**). The polarity reversal of ${I}_{Q}^{ph}$ is noticed at $\pm {V}_{0}$ and it is schematically presented in (**h**). For $V > |{V}_{0}|$, the dot dumps heat into the hotter reservoirs.

So far we noticed that charge transport manipulates the non-equilibrium ($\lambda \ne 0$) phonon distribution. However, it is evident from (14), that the phonon distribution finds a strong dependence on the temperature of reservoirs and hence we test the phonon distribution in three cases: (i) Case H: where, the temperature of the reservoirs is greater than the temperature of electrodes (${T}_{B}={T}_{BH} > T$), (ii) Case E: where, the temperature of the reservoirs is equal to the temperature of electrodes (${T}_{B}={T}_{BE}=T$) and (iii) Case C: where, the temperature of the reservoirs is less than the temperature of electrodes (${T}_{B}={T}_{BC} < T$). The inset of Fig. 4(b) shows that in Case H, ${N}_{ph}^{eq}=[exp(\frac{\hslash \omega }{{k}_{B}{T}_{BH}})-{\mathrm{1]}}^{-1}$ is greater than $\langle {N}_{ph}\rangle$ at short-circuit point (${V}_{SC}=0$). When voltage increases, $\langle {N}_{ph}\rangle$ shoots up and overcomes ${N}_{ph}^{eq}$ at $|{V}_{0}|$. Figure 4(b) notes that at $V=|{V}_{0}|$, the dot phonon distribution equals the equilibrium phonon distribution. In the Case E, $\langle {N}_{ph}\rangle$ equals with ${N}_{ph}^{eq}=[exp(\frac{\hslash \omega }{{k}_{B}{T}_{BE}})-{\mathrm{1]}}^{-1}$ at the short-circuit point (${V}_{SC}=0$) but in Case C, $\langle {N}_{ph}\rangle$ is always larger than ${N}_{ph}^{eq}=[exp(\frac{\hslash \omega }{{k}_{B}{T}_{BC}})-{\mathrm{1]}}^{-1}$ for all voltages. These aspects are pictorially shown in the insets of Fig. 4(c) and (d) respectively. Consequently, in Case E, the phonon distribution equals the equilibrium distribution at $V={V}_{SC}$ as noticed in Fig. 4(c). On the other hand, Fig. 4(d) shows that the dot phonons remain in a state of non-equilibrium for all voltages in Case C.

Next, we investigate the variation of non-equilibrium ${I}_{Q}^{ph}$ with voltage with ${I}_{Q}^{ph}$ renamed as ${I}_{QH}^{ph}$, ${I}_{QE}^{ph}$ and ${I}_{QC}^{ph}$ for Cases H, E and C respectively. Similarly, the non-equilibrium dot temperature ${T}_{M}$ is renamed as ${T}_{MH}$, ${T}_{ME}$ and ${T}_{MC}$ in these three cases. Figure 4(e) notes that ${I}_{QH}^{ph}$ reverses its polarity at $|{V}_{0}|$ and the setup dumps heat into the hotter reservoirs when $V > |{V}_{0}|$. However Fig. 4(f) and (g) do not depict any polarity reversal for ${I}_{QE}^{ph}$ and ${I}_{QC}^{ph}$. The voltage variation of ${I}_{QH}^{ph}$,${I}_{QE}^{ph}$ and ${I}_{QC}^{ph}$ are consistent with the voltage variation of ${T}_{MH}$, ${T}_{ME}$ and ${T}_{MC}$, depicted in the insets of Fig. 4(e),(f) and (g) respectively. The schematic of ${I}_{QH}^{ph}$ is separately presented in Fig. 4(h). The counter-intuitive scenario that the setup is dumping heat into a hotter reservoir is a unique outcome of the non-equilibrium condition ($\lambda \ne 0$).

### Charge induced phonon switching

**Figure 5:**
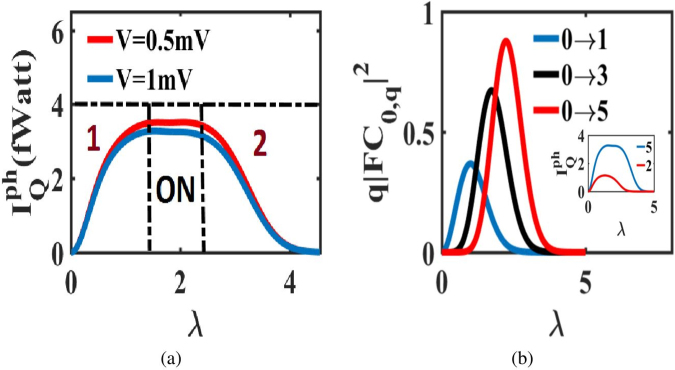
Phonon switch facilitated by *λ*: (**a**) Variation of ${I}_{Q}^{ph}$ with *λ* at fixed voltage. The profile gives an idea of the phonon switch with different operating regions. Region 1 and 2 are the operating regions when the switch is turned on and off respectively. The ON region is when the switch remains on. (**b**) Variations of different phonon generation processes with *λ* that control the switch operation. The inset plot shows how more number of phonon side-bands broaden the ON region and aid efficient switching.

We note that if one wishes to switch phonon currents, voltage is not a suitable control parameter because ${I}_{Q}^{ph}$ cannot even turn on when *λ* = 0. Even in the finite *λ* case, ${I}_{Q}^{ph}$ can be turned on but not turned off. However, switching of phonon currents can be achieved by varying *λ* at a constant voltage. In Fig. 5(a), we investigate the variation of ${I}_{Q}^{ph}$ as a function of *λ* at fixed voltage. The phonon current first shoots up, levels off and finally falls as *λ* is ramped up. Therefore, ${I}_{Q}^{ph}$ can be switched on and off via the modulation of *λ*. According to (12), the phonon generation rate associated with the charge transfer between two phonon states with phonon number *q*_1_ and *q*_2_ is proportional to (*q*_2_ _−_ *q*_1_) ${|F{C}_{{q}_{1},{q}_{2}}|}^{2}$. In Fig. 5(b), we plot the variation of $F{C}_{{q}_{1},{q}_{2}}^{2}$ as a function of *λ* for different values of *q*_2_ with *q*_1_ = 0. It is observed that for the small values of *λ*, $F{C}_{{q}_{1},{q}_{2}}^{2}$ shoots up and the switch is turned ON as we increase *λ*. As *λ* is increased further, $F{C}_{{q}_{1},{q}_{2}}^{2}$ starts to decay. The decay starts at earlier *λ* for smaller values of ${q}_{2}$ and shifts to larger values of *λ* with increasing ${q}_{2}$. At intermediate *λ*, ${I}_{Q}^{ph}$ levels off and the switch remains ON. At large *λ* all the processes fall sharply, and the switch is turned OFF. Recently, a pioneering demonstrated^20^ that *λ* can be turned ON and OFF in a small bandgap suspended carbon nanotube quantum dot by controlling the location of quantum dot along the tube by applying voltage through a series of gates. In addition to that, specific electronic and phonon modes can be coupled in real space. Theoretically this kind of selective coupling leads to the tuning of $\lambda$^21,35^. The electron-induced thermal switch that we have proposed can thus be accomplished using such kinds of experimental set ups.

Phonon computation is an emerging area that aims to convert waste heat (phonon currents in our case) to electrical signals which can be re-implemented in logic design. A major challenge in that course is to modulate the phonon current by electrical means. Our proposal for phonon switching can accomplish such a goal. This switching can be used to design thermal pulse generators and thermal multi-vibrators. For efficient switching, the ‘ON’ region should be more broadened, and that can be achieved by increasing the number of phonon sidebands in the voltage window as shown in the inset of Fig. 5(b). The incrementation of sidebands is feasible by reducing the frequency of phonon mode.

## Discussion

The non-linear transport regime in a quantum dot heat engine described by *Anderson-Holstein* model was investigated in detail. It was shown that a finite electron-phonon interaction leads to a charge induced phonon generation that stimulates a phonon current even in the absence of a thermal gradient. This gave rise to the non-linear phonon Peltier effect which increases with the electron-phonon interaction. Utilizing the manipulation of phonon distribution via charge induced phonon accumulation, we demonstrated that the polarity of the phonon heat current can be reversed so that setup can dump heat into hotter reservoirs. In further exploring possibilities that can arise from this effect, we proposed a charge-induced phonon switching mechanism as a building block for phonon computation.

## Methods

In this work, the evaluation of the probability ${P}_{n,q}$ of an electron-phonon many-body state $|n,q\rangle$, the charge current ${I}_{{\alpha }_{1}}$ and the phonon heat current ${I}_{{\alpha }_{2}}^{Q}$ is carried out using matrix computation. We enlist below the crucial steps that are necessary to be elucidated regarding the simulation method.

1. The parameters used are as follows. The on-site energy is set to $\varepsilon =1\,meV$, so that $\varepsilon  < 2{k}_{B}T$ ($T=10\,K$), which is a relevant for thermoelectric operations^25^. The Coulomb interaction *U* is set to 0.1 eV. We chose large *U* ($U\gg {k}_{B}T$) to avoid double occupancy in the dot within the allowed voltage range. The rate of electron tunneling ($\gamma ={10}^{-5}/\hslash$ ${S}^{-1}$) is set higher than the phonon relaxation rate ($\beta ={10}^{-6}/\hslash$ ${S}^{-1}$) to ensure that the phonon reservoirs can change the dot phonon number by ±1 when a single electron tunnels into or out of the dot. The phonon frequency is set higher than γ ($\hslash \omega  > \hslash \gamma$, $\omega =1.5\times {10}^{11}\,se{c}^{-1}$), to exclude the overlap between the neighboring phonon sidebands.
2. The steady state probability ${P}_{n,q}$ of $|n,q\rangle$ is evaluated from (9), using the condition $\sum _{n,q}{P}_{n,q}=1$. In this context, simultaneous algebraic equations are solved by matrix inversion method. Using ${P}_{n,q}$, we can derive the charge current ${I}_{{\alpha }_{1}}$ and the phonon current ${I}_{{\alpha }_{2}}^{Q}$ from (10) and (11) respectively.
3. In Fig. 2(a), ${I}_{Q}^{ph}$ is calculated considering all the reservoirs and electrodes at the same temperature (${T}_{H}={T}_{C}={T}_{L}={T}_{R}=10\,K$). The same operating condition is maintained in Fig. 3(a), where the voltage dependence of ${\pi }_{ph}$ is plotted.
4. The dot temperature *T*_*D*_ in Fig. 3(d) is estimated from a phonon thermometer bath. The estimation is based on the principle that the voltage dependent phonon current between the dot and thermometer vanishes at the temperature *T*_*D*_. While estimating the temperature, the phonon thermometer is weakly coupled to the dot as compared to the other reservoirs ($\hslash {\beta }_{T}={10}^{-15}$ eV and $\hslash {\beta }_{H(C)}={10}^{-6}$ eV) so that it does not interrupt the other transport processes.
5. The role of the temperature of reservoirs in controlling ${I}_{Q}^{ph}$ is examined in three cases. In Case H, the reservoirs are hotter than the electrodes (*T*_*BH*_ = 30 *K* and *T* = 10 *K*). In Case E, the reservoirs and electrodes are at the same temperature (*T*_*BE*_ = *T* = 10 *K*). In Case C, the reservoirs are colder than the electrodes (*T*_*BC*_ = 5 *K* and *T* = 10 *K*). In all the cases, ${I}_{Q}^{ph}$ is derived from (11).
